# Intraoperative Contrast‐Enhanced Ultrasound to Assess Lymph Node Resection and Involvement in Patients Undergoing Robotic‐Assisted Thoracic Surgery Lobectomy for Management of Non‐Small Cell Lung Cancer

**DOI:** 10.1111/1759-7714.70308

**Published:** 2026-09-27

**Authors:** Messina Gaetana, Morena Fasano, Davide Gerardo Pica, Giuseppe Vicario, Martina Robustelli, Vincenzo di Filippo, Francesca Capasso, Anna D'Agostino, Rosa Mirra, Beatrice Leonardi, Riccardo Vinciguerra, Alessia Angela Guarino, Ciro Balbo, Sara Di Lorenzo, Mariellina Laudato, Giovanni Vicidomini, Fortunato Ciardiello, Antonio D'Andrilli, Alfonso Fiorelli

**Affiliations:** ^1^ Thoracic Surgery Unit Università degli Studi della Campania “Luigi Vanvitelli”, Napoli Naples Campania Italy; ^2^ Precision Medical Department, Medical Oncology University of Campania “Luigi Vanvitelli” Naples Italy; ^3^ Thoracic Surgery Unit University “Federico II” Naples Campania Italy

**Keywords:** CEUS: contrast‐enhanced ultrasound, lobectomy, lymph node, RATS: robotic‐assisted thoracic surgery

## Abstract

**Background:**

Mediastinal staging is crucial in patients undergoing lung resection for cancer to plan further adjuvant. Current guidelines recommend resecting at least three mediastinal lymph node stations, including Group7. Herein, we evaluated the role of intraoperative Contrast‐enhanced ultrasound (CEUS) to guide lymph node resection in patients undergoing RATS lobectomy for management of non‐small cell lung cancer.

**Methods:**

It was a retrospective study including 24 consecutive patients underwent RATS lobectomy for management lung cancer. The hilar and mediastinal lymph node was firstly evaluated with a CEUS and then resected. A bolus of 2.4 mL SonoVue was injected into the antecubital vein and the perfusion was scanned continuously for at least 3 min. The dynamic image obtained was used to evaluate the mediastinal and hilar lymph nodal and guide the dissection. A radical lymph‐adenectomy was performed in all cases independently from the CEUS results.

**Results:**

We evaluated 144 lymph nodes with a CEUS during RATS lobectomy. Histological examination of the lymph nodes showed five lymph nodes metastatic (3.5%); of which 3 (60%) metastatic N1 lymph nodes; 2 (40%) metastatic N2 lymph nodes. Intraoperative CEUS of hilar and mediastinal lymph nodes showed: sensitivity 83%; specificity 98%; PPV, 77%; PNV, 99% and accuracy 94%.

**Conclusion:**

Our preliminary results seem to confirm the value of intraoperative CEUS to assess lymph node resection during RATS lobectomy. It may help the lymph node dissection and reduce the risk of intraoperative injury. Future study should confirm our preliminary results and confirm the value of intraoperative CEUS.

## Graphical abstract

Graphical abstract representing the main the study.

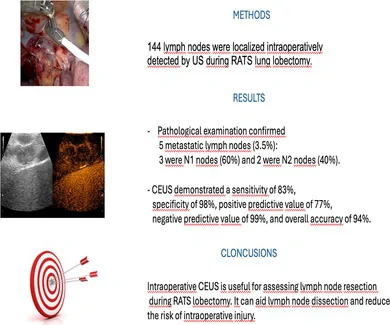

## 1 Introduction

Robotic‐assisted thoracic surgery (RATS) is now widley adopted worldwide as a surgical option for patients with non‐small cell lung cancer (NSCLC) [1, 2, 3, 4, 5]. Accurate mediastinal lymph node staging in patients undergoing pulmonary resection is essential for planning adjuvant therapy. Systematic lymph node dissection (SND) entails the removal of intrapulmonary, hilar, and mediastinal nodes according to well‐defined anatomical landmarks. In contrast, lobe‐specific lymph node dissection targets mediastinal nodes based on the site of the primary lesion. The status of N2 nodes plays a decisive role not only in shaping treatment strategies but also in influencing overall prognosis. Although radical lymphadenectomy is generally recommended, it carries the risk of postoperative complications. For this reason, current guidelines advise the removal of at least three mediastinal lymph node stations, including station 7. This underscores the need for intraoperative tools capable of guiding the surgeon in performing accurate lymph node dissection during lung resections [6, 7, 8, 9]. The present study investigates the potential role of intraoperative contrast‐enhanced ultrasound (Io‐CEUS) in lymph node dissection during robotic lobectomy for NSCLC [10, 11, 12, 13, 14].

## 2 Materials and Methods

This study included patients with NSCLC who underwent lobectomy with lymphadenectomy using RATS combined with intraoperative CEUS at the Thoracic Surgery Unit of the University of Campania “Luigi Vanvitelli” between January 2024 and March 2025. All patients provided informed consent after a preoperative explanation of the procedure, and the study received approval from the institutional ethics committee. Surgery was carried out under general anesthesia with single‐lung ventilation, with patients placed in the lateral decubitus position. Pleural cavity expansion was achieved using CO_2_ insufflation (5–8 mmHg). The Da Vinci XI robotic platform was positioned posterior to the patient, with four trocars inserted between the 7th and 8th intercostal spaces. The ultrasound system was connected to the robotic console through a DVI cable, with access authorized by the operating surgeon. A linear L51K probe (2–12 MHz), compatible with the Da Vinci system, was used. The 26 mm probe, introduced by the assistant through a thoracostomy port, had a flat tip to ensure full contact with mediastinal structures at any angle, allowing wide freedom of maneuver [8, 15, 16, 17, 18]. Sterile saline was applied to reduce acoustic impedance, enabling high‐resolution imaging (Figure 1). Insonation sites varied depending on the side of the chest: Left side: bordered anteriorly by the phrenic nerve, posteriorly by the vagus nerve, superiorly by the aortic arch, and caudally by the left main bronchus. Right side: bordered superiorly by the innominate artery, anteriorly by the superior vena cava, posteriorly by the trachea, and inferiorly by the right upper lobe [19, 20, 21, 22, 23]. Suspicious intrapulmonary, hilar, and mediastinal nodes were assessed with CEUS (Figure 2). Contrast consisted of 2.4–5 mL of sulfur hexafluoride microbubbles (SonoVue) administered via central venous catheter, followed by a 10–20 mL saline flush. SonoVue microbubbles, coated with phospholipids, undergo shape and size variations as they circulate through the microvasculature. Node perfusion was observed in real time for the first 60 s and then re‐evaluated at 120, 180, 240, and 300 s (Figure 3). Two imaging phases were defined: early phase (0–120 s): assessment of filling times, enhancement pattern (heterogeneous, punctate, annular, patchy), and detection of non‐enhanced areas or artifacts and the late phase (> 120 s), for the evaluation of contrast washout and its duration. Key findings such as perfusion defects and interruption of the contrast “ring” were considered significant for identifying metastatic nodes. This dynamic imaging allowed real‐time selection of suspicious mediastinal and hilar nodes, followed by guided resection. Nonetheless, radical lymphadenectomy was systematically performed in all patients regardless of CEUS results [24, 25, 26, 27, 28]. Excised nodes considered malignant by B‐mode or CEUS were marked with Vicryl sutures and sent for histopathological confirmation (Figure 4). A chest X‐ray was obtained on postoperative day one and again before chest tube removal. The first multidisciplinary follow‐up visit, involving thoracic surgeons and oncologists, was scheduled 1 month after surgery [10, 29, 30, 31, 32, 33].

**FIGURE 1:**
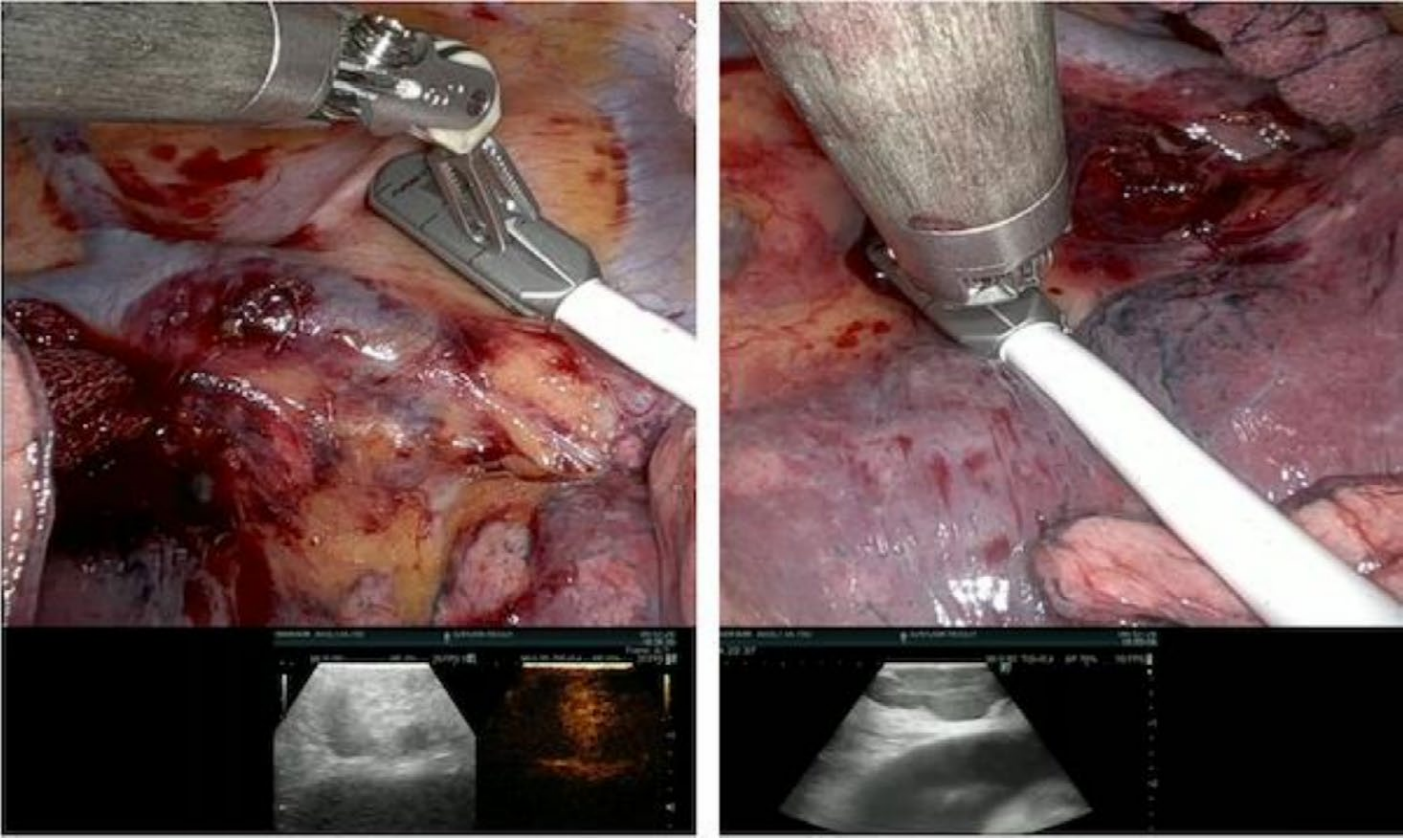
The 26 mm probe, introduced by the assistant through a thoracostomy port, had a flat tip to ensure full contact with mediastinal structures at any angle, allowing wide freedom of maneuver.

**FIGURE 2:**
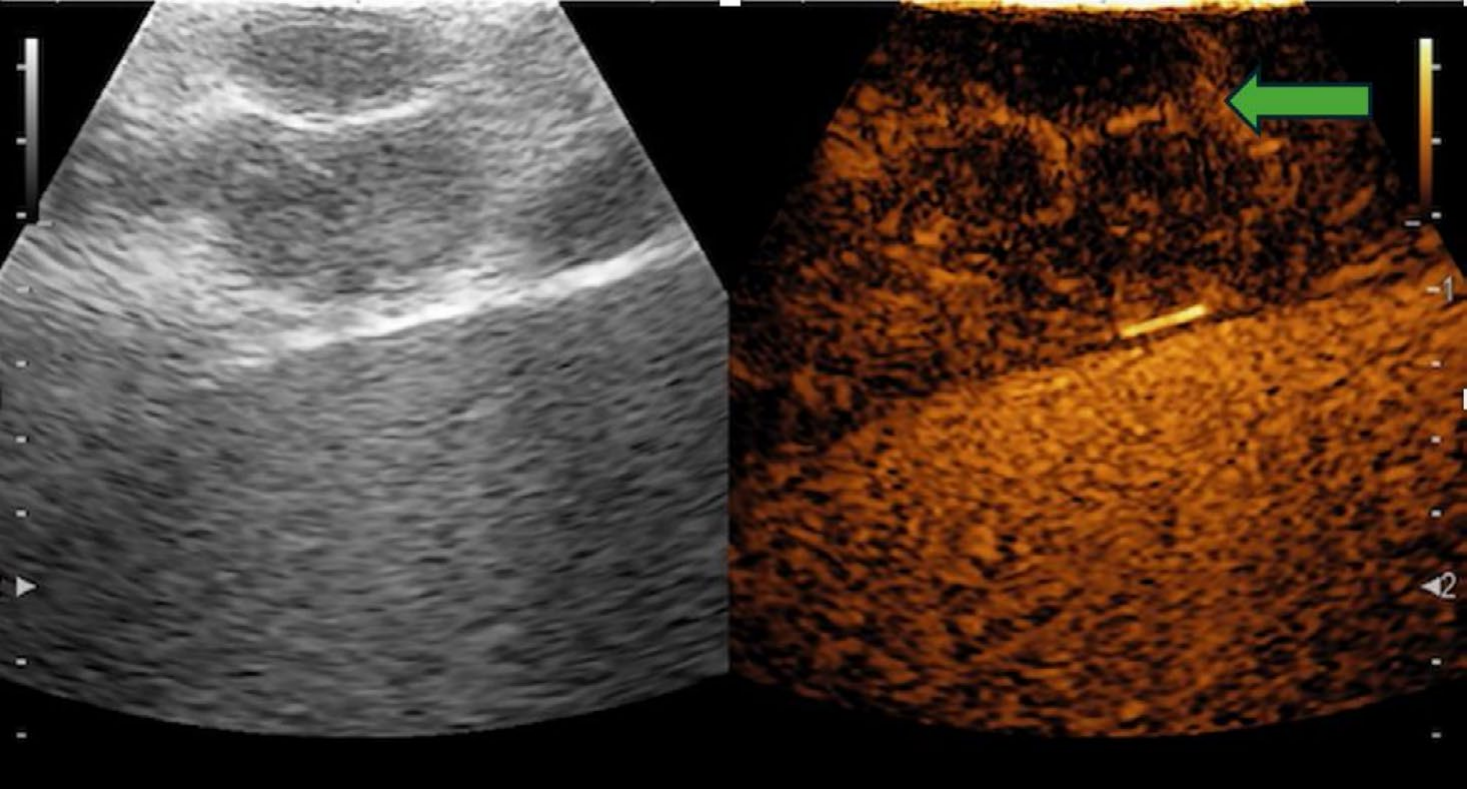
Suspicious intrapulmonary, hilar, and mediastinal nodes were assessed with CEUS.

**FIGURE 3:**
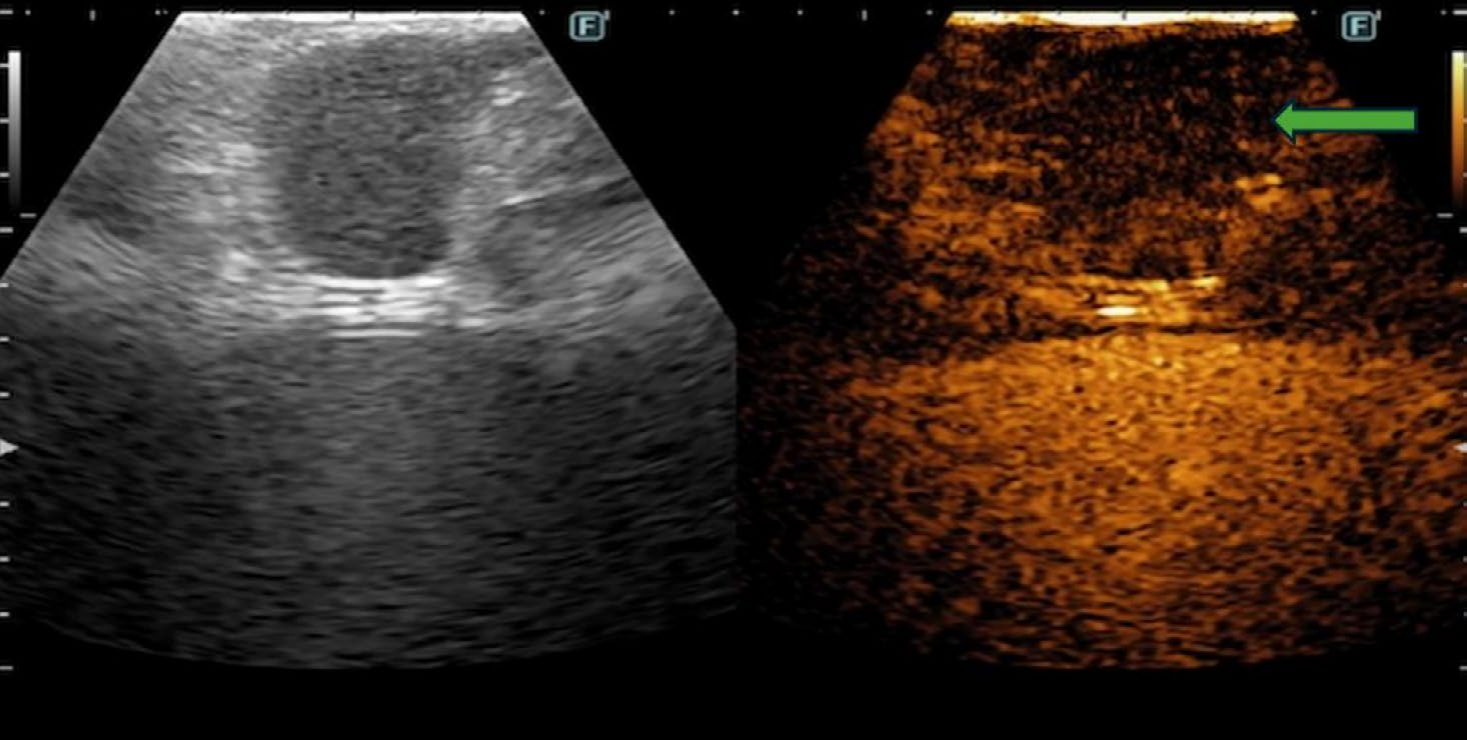
Sonovue perfusion was observed in real time.

**FIGURE 4:**
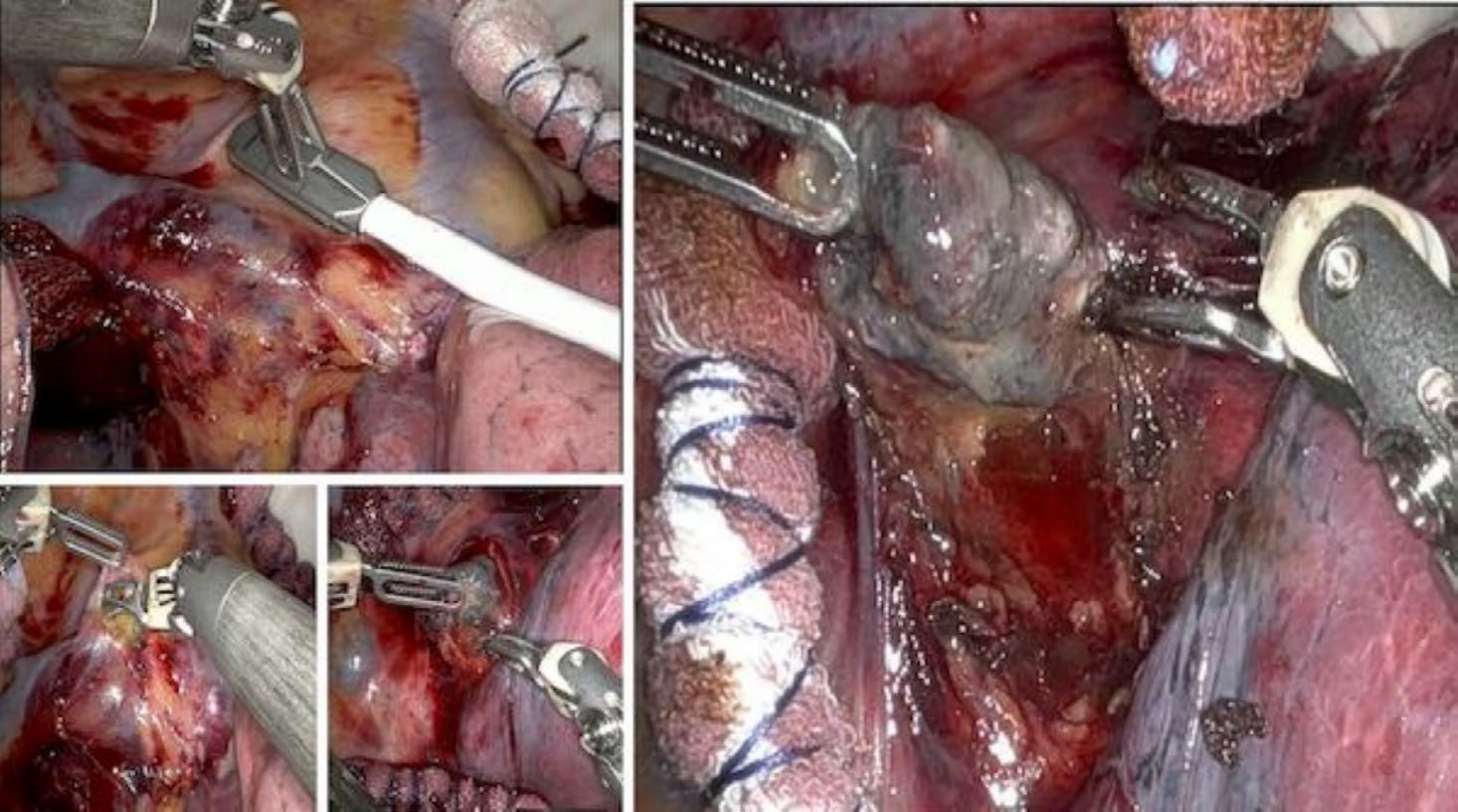
This dynamic imaging allowed real‐time selection of suspicious mediastinal and hilar nodes, followed by guided resection.

### 2.1 Statistical Analysis

We evaluated 144 lymph nodes with a CEUS during RATS lobectomy. Histological examination of the lymph nodes showed five lymph nodes metastatic (3.5%); of which three (60%) metastatic N1 lymph nodes; two (40%) metastatic N2 lymph nodes. Intraoperative CEUS of hilar and mediastinal lymph nodes showed: sensitivity, 83%; specificity, 98%; PPV, 77%; PNV, 99%, and accuracy, 94% (Figure 5).

**FIGURE 5:**
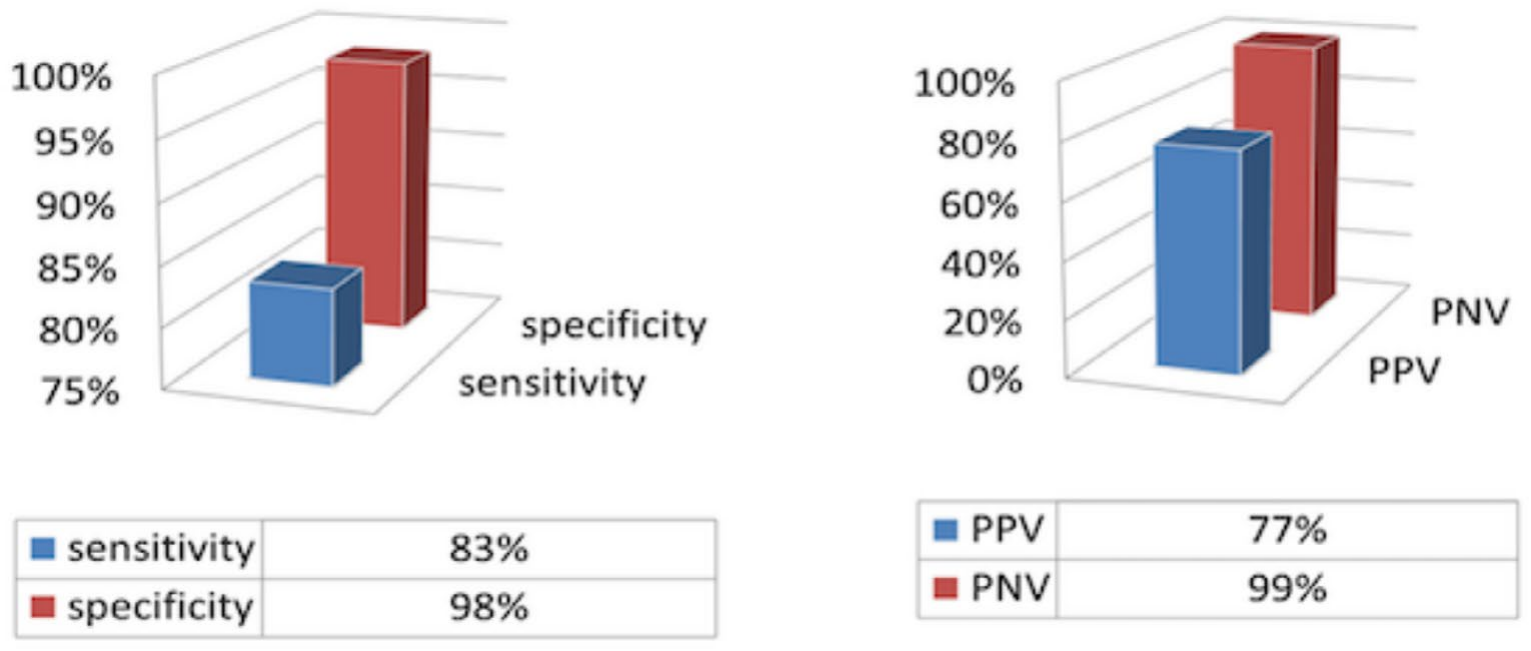
Statistical analysis.

## 3 Results

This retrospective study included 24 consecutive patients who underwent robotic‐assisted lobectomy for NSCLC at the Thoracic Surgery Unit of the University of Campania “Luigi Vanvitelli.” Preoperative assessment included whole‐body CT scan, FDG‐PET, blood chemistry, chest imaging, ECG, and pulmonary function tests. Patients with T1–3, N0, M0 disease were enrolled. The exclusion criteria related to the use of CEUS were hypersensitivity to sulfur while those related to surgery were severe pulmonary hypertension, respiratory failure, ischemic heart disease, and pregnancy. None of the 24 patients who underwent CEUS had received neoadjuvant therapy. All patients received intraoperative CEUS for mediastinal lymph node assessment. The mean patient age was 65 years (range …). Tumor location was as follows: right upper lobe (eight patients, 33.3%), left lower lobe (five patients 20.83%), left upper lobe (four patients, 16.7%), right lower lobe (five patients, 20.83%), and middle lobe (two patients, 8.33%). Histological types included 13 adenocarcinomas, six squamous cell carcinomas, and five adenosquamous carcinomas. Surgical procedures consisted of eight right upper lobectomies, five right lower lobectomies, five left lower lobectomies, four left upper lobectomies, and two middle lobectomies. Intraoperative B‐mode ultrasound findings included: microcalcifications (13 patients, 54.17%), hyperechogenicity (16 paiets, 66.7%), cystic areas (11 patients, 45.83%), absence of a visible hilum (24 patients, 100%), and peripheral vascularization (21 patients, 87,5%). CEUS analysis showed heterogeneous enhancement (punctate, patchy, or annular) within the first 10–15 s in 22 patients; absence of a linear hyperechogenic vascular image in 23 patients; and early washout (< 120 s) in all 24 patients. A total of 144 lymph nodes were evaluated with CEUS. Pathological examination confirmed 5 metastatic nodes (3.5%): three were N1 nodes (60%) and two were N2 nodes (40%). CEUS demonstrated a sensitivity of 83%, specificity of 98%, a positive predictive value of 77%, negative predictive value of 99%, and an overall accuracy of 94%. The five metastatic lymph nodes confirmed by histology corresponded to the lymph nodes considered malignant by intraoperative CEUS. The performance of CEUS was not affected by the location of the different lymph node stations. Minor postoperative complications related to lymphadenectomy were lymphorrhea and minor bleeding while other complications not directly related to lymphadenectomy included persistent air leakage (> 5 days) found in 6 patients. Postoperative pain management included locoregional nerve blocks, and all patients were treated following an Enhanced Recovery After Surgery (ERAS) protocol. Nineteen patients were correctly staged using CEUS. Follow‐up visits were scheduled every 3 months for 3 years, then every 6 months for two additional years (Table 1).

**TABLE 1:** Patient characteristics.

Category	Subgroup	*N*	%
Age	Mean	65 years	—
Pulmonary nodule location	RUL (right upper lobe)	8	33.33
	LLL (left lower lobe) LUL (left upper lobe) RLL (right lower lobe) ML (middle lobe)	5 4 5 2	20.83 16.67 20.83 8.33
Histology	Squamous cell carcinoma	6	25
	Adenocarcinoma Adenosquamous carcinoma	13 5	54.17 20.83
Surgical resections	RLL lobectomy (right lower)	5	20.83
	RUL lobectomy (right upper) LLL lobectomy (left lower) LUL lobectomy (left upper) ML lobectomy (middle)	8 5 4 2	33.33 20.83 16.67 8.33
Intraoperative US of lymph nodes	Microcalcifications Hyperechogenicity Cystic areas Absence of hilum Peripheral vascularization	13 16 11 24 21	54.17 66.67 45.83 100 87.5
Intraoperative CEUS of lymph nodes	Heterogeneous enhancement (spots, punctate, ring‐shaped, 10–15 s) Absence of linear hyperechoic image (pulmonary artery enhancement) Rapid washout (< 120 s)	22 23 24	91.67 95.83 100
Lymph node data	Total metastatic lymph nodes N1 metastatic N2 metastatic	144 5 3 2	4.38 60 (of metastatic) 40 (of metastatic)

## 4 Discussion

Robotic surgery is experiencing rapid worldwide adoption. Current evidence suggests that robotic‐assisted thoracoscopic surgery provides outcomes comparable in safety and efficacy to video‐assisted thoracoscopic surgery (VATS) [30, 34, 35]. However, the oncological completeness of mediastinal lymph node dissection performed with RATS remains debated. Some studies argue that RATS is not superior to VATS in terms of nodal yield or upstaging, while others report that robotic procedures may enable more extensive lymph node dissection and higher rates of nodal upstaging. The European Society of Thoracic Surgery (ESTS) advocates radical lymphadenectomy, including systematic dissection of stations 2R/L, 4R/L, 10R/L, and 7, and extending to Stations 5, 6, 8, and 9 [36, 37]. Other studies, particularly from East Asia, support lobe‐specific lymphadenectomy in selected cases (e.g., cT1N0 NSCLC), finding no significant differences in postoperative complications or survival compared with systematic dissection [23, 38, 39]. The Japanese Clinical Oncology Group (JCOG) has further demonstrated that systematic lymphadenectomy carries a higher risk of complications compared to lobe‐specific approaches. Various techniques have been tested to improve intraoperative nodal mapping, including technetium‐99 m injection, blue dye, and Doppler ultrasound, but none has achieved widespread acceptance due to limitations in accuracy or visualization. In this context, CEUS offers unique advantages: it can detect microvessels smaller than 100 μm, provides real‐time information on nodal perfusion, and does not expose patients to radiation. In our series, two CEUS signs—perfusion defects and interruption of the peripheral contrast ring—proved highly suggestive of metastatic involvement. These findings are consistent with tumor‐driven angiogenesis, characterized by irregular vascular networks and arteriovenous shunts. CEUS also benefits from being minimally invasive, repeatable, and relatively low‐cost compared with other imaging techniques [40, 41, 42, 43]. Importantly, integration with robotic technology enhances CEUS performance: the robotic arm offers greater maneuverability of the ultrasound probe, precise angulation, and superior visualization of mediastinal anatomy. This combination facilitates accurate identification of metastatic nodes with minimal impact on operative time, while maintaining safety and reproducibility. The main limitation of CEUS remains the lack of panoramic imaging and the need for operator expertise in intra‐thoracic ultrasound [44, 45, 46, 47, 48].

## 5 Conclusions

RATS is an evolving surgical platform, and its integration with CEUS has the potential to significantly enhance the precision and effectiveness of mediastinal lymph node dissection. The real‐time vascular characterization provided by CEUS, combined with the superior dexterity and visualization of robotic systems, allows for more reliable identification and targeted resection of suspicious nodes. This combined approach demonstrates high safety, reproducibility, and diagnostic accuracy, and could become the preferred strategy for mediastinal lymphadenectomy in appropriately selected NSCLC patients. While systematic lymph node dissection remains the standard for early‐stage NSCLC, CEUS‐guided nodal assessment during RATS may open the way to more tailored surgical strategies—particularly in elderly or comorbid patients where segmentectomy and lobe‐specific lymphadenectomy could represent safer alternatives.

Further multicenter clinical trials will be necessary to validate these findings and to better define the role of CEUS in intraoperative staging of NSCLC.

## Author Contributions

**Messina Gaetana:** conceptualization, writing – review and editing, writing – original draft. **Giuseppe Vicario:** methodology. **Davide Gerardo Pica:** methodology. **Rosa Mirra:** software. **Vincenzo di Filippo:** data curation, investigation. **Martina Robustelli:** data curation, validation. **Sara Di Lorenzo:** methodology, investigation. **Morena Fasano:** conceptualization, writing – review and editing, writing – original draft. **Beatrice Leonardi:** investigation. **Alessia Angela Guarino:** data curation. **Riccardo Vinciguerra:** methodology. **Francesca Capasso:** investigation. **Anna D'Agostino:** software. **Alfonso Fiorelli:** supervision. **Ciro Balbo:** methodology, investigation. **Fortunato Ciardiello:** supervision. **Antonio D'Andrilli:** supervision. **Mariellina Laudato:** investigation, methodology. **Giovanni Vicidomini:** supervision.

## Funding

This work was supported by University of Campania “L. Vanvitelli”.

## Ethics Statement

The authors are accounle for all aspects of the work in ensuring that questions related to the accuracy or integrity of any part of the work are appropriately investigated and resolved. The study was led in compliance with the principles of the Declaration of Helsinki; written informed consent was obtained from all participants during preoperative communication and the protocol was approved by the Ethics Committee of the University of ‘Luigi Vanvitelli’ of Naples (18 296/2025).

## Conflicts of Interest

The authors declare no conflicts of interest.

## Data Availability

The data that support the findings of this study are available from the corresponding author upon reasonable request.
